# SERCA Activity Controls the Systolic Calcium Increase in the Nucleus of Cardiac Myocytes

**DOI:** 10.3389/fphys.2019.00056

**Published:** 2019-02-06

**Authors:** Tobias-Oliver Kiess, Jens Kockskämper

**Affiliations:** Institute of Pharmacology and Clinical Pharmacy, Biochemical and Pharmacological Center, University of Marburg, Marburg, Germany

**Keywords:** nuclear calcium, cardiac myocyte, calcium signaling, SERCA, nuclear envelope (NE)

## Abstract

In cardiomyocytes, nuclear calcium is involved in regulation of transcription and, thus, remodeling. The cellular mechanisms regulating nuclear calcium, however, remain elusive. Therefore, the aim of this study was to identify and characterize the factors that regulate nuclear calcium in cardiomyocytes. We focused on the roles of (1) the cytoplasmic calcium transient (CaT), (2) the sarcoplasmic/endoplasmic reticulum calcium ATPase (SERCA), and (3) intracellular calcium stores for nuclear calcium handling. Experiments were performed on rat ventricular myocytes loaded with Fluo-4/AM. Subcellularly resolved CaTs were visualized using confocal microscopy. The cytoplasmic CaT was varied by reducing extracellular calcium (from 1.5 to 0.3 mM) or by exposure to isoprenaline (ISO, 10 nM). SERCA was blocked by thapsigargin (5 μM). There was a strict linear dependence of the nucleoplasmic CaT on the cytoplasmic CaT over a wide range of calcium concentrations. Increasing SERCA activity impaired, whereas decreasing SERCA activity augmented the systolic calcium increase in the nucleus. Perinuclear calcium store load, on the other hand, did not change with either 0.3 mM calcium or ISO and was not a decisive factor for the nucleoplasmic CaT. The results indicate, that the nucleoplasmic CaT is determined largely by the cytoplasmic CaT via diffusion of calcium through nuclear pores. They identify perinuclear SERCA activity, which limits the systolic calcium increase in the nucleus, as a novel regulator of the nuclear CaT in cardiac myocytes.

## Introduction

Calcium (Ca) is a ubiquitous ion and versatile second messenger throughout all types of cells. It regulates a variety of important cellular processes, e.g., electrical signaling, metabolism, secretion, gene transcription and cell cycle (Berridge et al., 2000). In cardiac muscle, Ca constitutes a key player in excitation-contraction coupling (ECC), linking electrical stimulation to mechanical contraction. During each heartbeat free intracellular Ca concentration rises to a systolic level of ∼1 μM enabling interaction between the contractile filaments, followed by Ca removal to a diastolic level of ∼100 nM causing relaxation (Bers, 2001; Kockskämper, 2016). Besides the well characterized functions of cytosolic Ca in driving the contraction-relaxation cycle in ECC, more recent studies have revealed the role of Ca in controlling gene transcription in cardiac myocytes in a process termed excitation-transcription coupling (ETC) (Wu et al., 2006; Dewenter et al., 2017). Moreover, evidence has accumulated that expression patterns of Ca-regulating proteins are not fixed but rather in a constant state of remodeling controlled by Ca-mediated ETC in both the healthy and diseased heart. For the adult heart it is known, that in response to neurohormonal and mechanical stress ETC can induce the re-expression of a fetal gene program, resulting in maladaptive hypertrophy and remodeling of ion channels and transporters, ultimately impairing cardiac function (Domínguez-Rodríquez et al., 2012; Dewenter et al., 2017).

The two pro-hypertrophic transcription factors GATA4 and myocyte enhancer factor 2 (MEF2) are activated by two common Ca-dependent signaling pathways: (1) the Ca/calmodulin-dependent protein phosphatase calcineurin-NFAT-GATA4/6 pathway (Wilkins et al., 2004) and (2) the Ca/calmodulin-dependent protein kinase II (CaMKII)-HDAC-MEF2 pathway (Wu et al., 2006). Both mechanisms are activated by elevated Ca/CaM formation and subsequent signaling.

The difficulty in understanding how Ca controls cardiac hypertrophy is due to the fact, that cardiac myocytes are continuously subjected to large periodic Ca signals flooding through the cytoplasm and nucleus during each contraction. Alterations in the spatio-temporal properties, duration and amplitude of individual cytoplasmic Ca transients (CaT) produce subtle changes that might be integrated over time to stimulate a certain gene program involving pro-hypertrophic genes (Berridge et al., 2003). Thus, Ca activates contraction in a beat-to-beat manner during EC coupling, while it is also able to control transcription via ET coupling on a longer time scale (Maier and Bers, 2002).

The nucleus is surrounded by the nuclear envelope (NE), a membrane system composed of an inner and outer phospholipid bilayer with a lumen in between. While the inner nuclear membrane faces the nucleoplasm, the outer nuclear membrane extends into the cytoplasm to form the sarcoplasmic reticulum (SR). This results in a continuous lumen of the SR and the NE, making the NE a functional Ca storage compartment (Lee et al., 1998; Wu and Bers, 2006). Furthermore, various Ca-handling proteins are present at the NE including Ca release channels (ryanodine receptors, RyRs, and IP_3_ receptors) and Ca re-uptake proteins (SERCA, phospholamban and Na/Ca exchanger, NCX) (Gerasimenko and Gerasimenko, 2004; Ledeen and Wu, 2007; Zima et al., 2007; Escobar et al., 2011; Plačkić et al., 2016; Wu et al., 2016; Chen et al., 2018). This suggests, that the nucleus may have its own Ca signaling system, in parallel to the one in the cytoplasm.

Although the NE is defining the nucleus as an autonomous cellular compartment, it is penetrated by nuclear pore complexes (NPCs), which constitute cylindrical channels accompanied by an array of proteins controlling bidirectional transport of cargo between the cytoplasm and the nucleus. Molecules larger than ∼40 kDa need to be transported in an active, signal-dependent manner (Davis, 1995). The pores allow passive diffusion of molecules <5 nm in diameter, making them freely permeable for ions (including Ca) and other small molecules to diffuse along their concentration gradients into or out of the nucleus (Keminer and Peters, 1999; Bootman et al., 2009).

While alterations in cytoplasmic Ca handling have been clearly implicated in the pathogenesis of hypertrophy and heart failure (HF), nucleoplasmic Ca signaling, on the other hand, has been studied much less in this regard. Nuclear Ca levels are directly involved in ET coupling. The known Ca-dependent targets that initiate the pro-hypertrophic cascades (CaMKII and calcineurin) are found in the nucleus (Heineke and Molkentin, 2006; Olivares-Florez et al., 2018). Hence, nuclear Ca may be suspected to play a crucial role in the pathogenesis of cardiac hypertrophy and ultimately HF. Moreover, recent studies have shown, that during development of hypertrophy and HF specific alterations to the nuclear structure and function may occur including alterations of nuclear size, of number and density of NE invaginations and of perinuclear expression levels of receptors and channels involved in Ca homeostasis, such as IP_3_ receptors, RyRs and SERCA (Ljubojevic et al., 2014; Plačkić et al., 2016; Olivares-Florez et al., 2018).

Despite their importance for ET coupling, the cellular mechanisms regulating nucleoplasmic Ca are only poorly understood. Therefore, the aim of the present study was to identify and characterize the mechanisms regulating nuclear Ca. In particular, we focused on the roles of cytoplasmic Ca, the NE Ca stores and SERCA function as factors potentially involved in the regulation of nucleoplasmic Ca in cardiac myocytes.

## Materials and Methods

### Animal Model

Male Wistar-Kyoto rats (WKY) at 12–18 weeks of age were used for cell isolation, providing a standard model for adult healthy cardiac myocytes. Animals were administered an isoflurane anesthesia followed by decapitation.

All experimental procedures involving the use of animals were in agreement with European Union Council Directive 2010/63/EU as well as with the German Animal Welfare Act (Tierschutzgesetz). The study was approved by the local animal welfare authorities (V 54 – 19 c 20 15 (1) MR 20/29 Nr. A 21/2010, AK-9-2014-Kockskämper).

### Isolation of Ventricular Myocytes

Ventricular myocytes were isolated as described previously (Plačkić and Kockskämper, 2018). Briefly, upon sacrificing the animal, the heart was extracted rapidly and placed in ice-cold cardioplegic solution for further preparation. Following a standard Langendorff protocol, a cannula was inserted into the aorta and secured in place with a ligature, enabling to flush the vessels and to connect the heart to a perfusion system. A peristaltic pump-driven perfusion system provided a continuous flow for retrograde perfusion, which was used to digest the tissue by applying a modified Tyrode’s solution containing collagenase (CLS-2, Worthington, United States) and type XIV protease (Sigma Aldrich, Germany). After sufficient digestion the atria were removed from the heart and the remaining ventricular tissue was cut and dispersed yielding a cell suspension of ventricular myocytes. Cells were gradually adapted to a final Ca concentration of 1.5 mM and plated on laminin (50 μg/ml)-coated glass-bottomed culture dishes.

### Imaging of Nucleoplasmic and Cytoplasmic CaTs

Isolated ventricular myocytes were loaded with the cell permeable fluorescent Ca indicator Fluo-4/AM (8 μM) for 30 min. After wash out, cells were given another 20 min to allow for de-esterification. Subcellularly resolved CaTs were visualized using a confocal microscope (LSM510, Carl Zeiss, Germany) in line-scan mode. Setting the scan line traversing the nucleus enabled simultaneous recording of cytoplasmic and nucleoplasmic CaTs as described previously (Plačkić et al., 2016). Recorded time series consisted of 1,600 one-directional lines with a duration of 3.07 ms each, at a depth of 12 Bit. Confocality was achieved with the pinhole set to 140 μm leading to a confocal plane of 1.0 μm thickness. An argon laser was used to generate an excitation wavelength of 488 nm. Fluorescence emission was collected at >505 nm.

Myocytes were bathed in recording buffer, a modified Tyrode’s solution containing (mM): 140 NaCl, 5 KCl, 1.5 CaCl_2_, 0.5 MgCl_2_, 10 HEPES, 10 glucose, pH 7.4. Electrical field stimulation (40 V) was applied via two platinum electrodes pacing cells at 1 Hz frequency at room temperature. Isoprenaline (ISO, 10 nM) and thapsigargin (TG, 5 μM) were used to study the effects of β-adrenergic stimulation and SERCA inhibition, respectively. Caffeine (10 mM) was used to estimate Ca load of the SR and perinuclear stores (PN).

Fluorescence data analysis was performed in ImageJ (version 1.48). Fluorescence traces were background subtracted and smoothed once. From each time series a representative CaT was chosen for single transient analysis. For characterisation of CaTs we focused on four parameters: in order to estimate changes in Ca levels we analyzed diastolic calcium (F_0_), representing the baseline fluorescence signal, and systolic calcium (F), corresponding to the peak signal. Both were normalized to resting fluorescence (F_rest_), which was obtained in the absence of electrical stimulation. For a comparison of the CaT kinetics we calculated the time to peak (TTP) and the time constant tau of decay (τ), assuming the signal decline to follow a mono-exponential decay.

### Statistics

Data are expressed as mean ± SEM for *n* myocytes from *N* animals. Statistical analyses were performed using GraphPad Prism (GraphPad Software Inc., San Diego, United States). Wilcoxon signed rank test was used for within-group comparisons. When more than two groups were compared, we used a one-way ANOVA with Dunnett’s *post-hoc* test or Kruskal–Wallis test with Dunn’s *post-hoc* test for multiple comparisons. A value of *P* < 0.05 was considered a statistically significant difference. Correlation analysis was performed using Spearman’s test. Correlation coefficient *r* was considered to deviate significantly from 0 if *P* < 0.05. In case of significant correlation between two parameters, a linear regression analysis was conducted.

## Results

### Reduction of Extracellular Ca Concentration Decreases Nuclear CaTs

**FIGURE 1:**
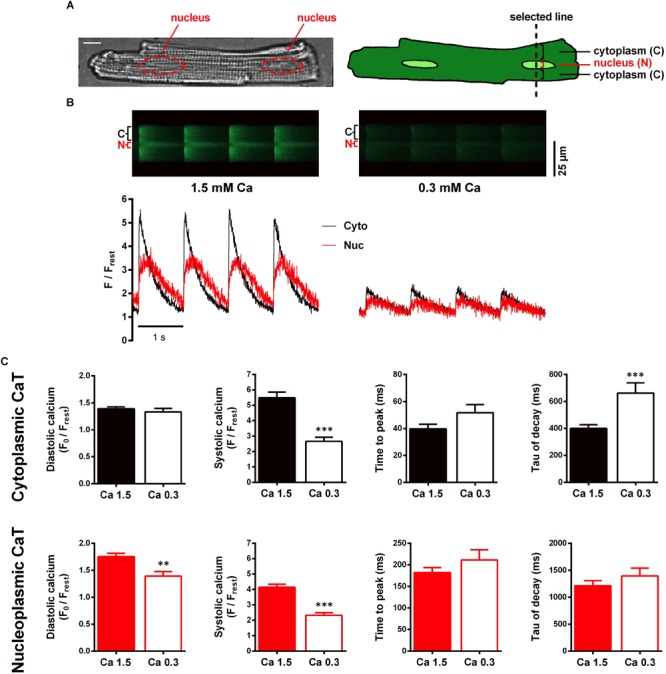
Characterisation of electrically stimulated cytoplasmic and nucleoplasmic CaTs in WKY ventricular myocytes at 1.5 and 0.3 mM extracellular Ca concentration. **(A)** Microscopic image of a ventricular myocyte with two nuclei highlighted in red; white scale bar represents 10 μm (left). Schematic drawing of a myocyte illustrating the scan line setting and the scanned regions (right). **(B)** Different myocyte. Left panel: original line-scan recording, showing electrically evoked cytoplasmic (C, Cyto, black) and nucleoplasmic (N, Nuc, red) CaTs at 1.5 mM extracellular [Ca] and the corresponding normalized fluorescence trace. Right panel: line-scan fluorescence image and corresponding trace of CaTs at 0.3 mM extracellular [Ca]. **(C)** Mean values of cytoplasmic and nucleoplasmic CaT parameters: diastolic Ca (F_0_/F_rest_), systolic Ca (F/F_rest_), time to peak and tau of decay. ^∗∗^*P* < 0.01, ^∗∗∗^*P* < 0.001, *n*/*N* = 19/7.

After loading with Fluo-4/AM the cells were superfused with recording buffer containing 1.5 mM Ca, which is referred to as control conditions. For subcellular Ca imaging line-scan confocal microscopy was used. The scan line was placed perpendicular to the longitudinal axis of the cell (Figure 1A), cutting through a nucleus. In this way the laser excites the nuclear region in the middle of the scan line, flanked by a cytoplasmic region above and below. Choosing this particular setting allowed us to obtain Fluo-4 fluorescence signals from cytoplasm and nucleus simultaneously. The left panel of Figure 1B displays an original time series of a line-scan under control conditions in which cyto- and nucleoplasmic CaTs were electrically evoked at 1 Hz stimulation. Increases in fluorescence brightness represent increases of Ca concentration. In the line-scan image the nucleoplasmic fluorescence signal (N, Nuc, red) can be distinguished from the cytoplasmic signal (C, Cyto, black) as it appears with a delay and as it exhibits a slower decay. This is also evident from the corresponding normalized fluorescence trace (F/F_rest_) below.

Our first aim was to elucidate the role of cytoplasmic Ca as a factor that may modulate the nuclear CaT. For this purpose, we performed interventions to increase and decrease the cytoplasmic CaT, respectively. This allowed us to investigate nuclear CaT regulation over a wide range of cytoplasmic Ca concentrations. To characterize the effect of a decreased cytoplasmic CaT on the nuclear CaT we exposed the cells to a low extracellular Ca concentration of 0.3 mM. Reduction of extracellular Ca provides a tool that can be used to modulate cytoplasmic Ca handling while interfering with intracellular signal pathways at a minimal level.

Switching the superfusion solution after basal recordings to a recording buffer containing 0.3 mM Ca (Ca 0.3) for 4–7 min allowed the CaTs to reach a new steady-state. CaTs impressively decreased in response to this treatment as depicted in the right panel of Figure 1B. Comparing the average results (Figure 1C), systolic calcium in cyto- and nucleoplasmic CaTs both significantly decreased to roughly 50% of the initial control (49% in cyto, from 5.49 ± 0.38 to 2.67 ± 0.26 F/F_rest_, *P* < 0.001, and 56% in nuc, from 4.14 ± 0.19 to 2.32 ± 0.17 F/F_rest_, *P* < 0.001; *n*/*N* = 19/7). However, a decrease in diastolic calcium could only be observed for the nucleus (from 1.75 ± 0.06 to 1.39 ± 0.08 F_0_/F_rest_, *P* < 0.01; *n*/*N* = 19/7). Comparing the kinetics, TTP increased in both compartments, but the changes were not statistically significant (cyto, from 40 ± 3 to 52 ± 6 ms, *P* = 0.066, and nuc, from 182 ± 12 to 211 ± 24 ms, *P* = 0.213; *n*/*N* = 19/7). The time constant τ, however, showed a significant increase in the cytoplasm (by 65% from 401 ± 27 to 662 ± 75 ms, *P* < 0.001) but not in the nucleus (from 1,212 ± 96 to 1,394 ± 147 ms, *P* = 0.361; *n*/*N* = 19/7). These results show, that systolic Ca in the nucleus decreases in response to reduced systolic Ca in the cytoplasm, suggesting that the systolic nucleoplasmic CaT follows the systolic cytoplasmic CaT, presumably by less passive Ca diffusion through nuclear pores.

### Modulation of Nuclear Ca Handling by Stimulation of β-Adrenergic Receptors

Next, we investigated the effects of increased cytoplasmic Ca levels on nuclear Ca handling. For that purpose, ISO (10 nM) was used to increase cAMP and Ca signaling by stimulation of β-adrenergic receptors.

**FIGURE 2:**
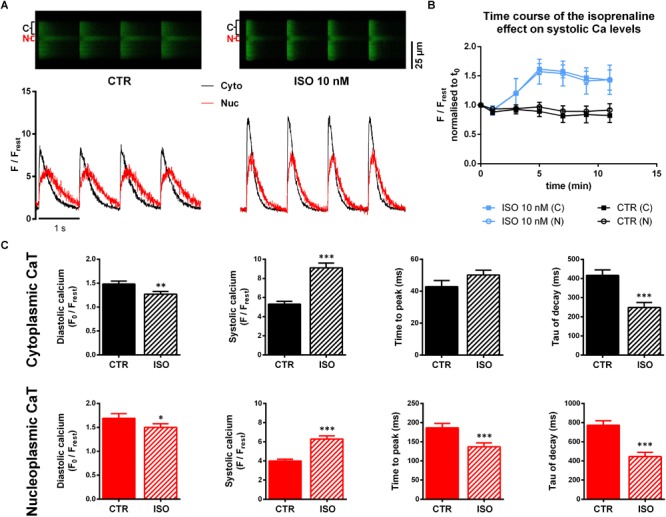
Confocal imaging of cytoplasmic and nucleoplasmic CaTs under isoprenaline (ISO) stimulation. **(A)** Left panel: original line-scan registration, showing electrically stimulated cytoplasmic (C, Cyto, black) and nucleoplasmic (N, Nuc, red) CaTs under CTR conditions and the corresponding normalized fluorescence trace. Right panel: line-scan fluorescence image and normalized trace after 5 min of ISO (10 nM) exposure. **(B)** Pilot experiments (*n*/*N* = 3/3 ISO; *n*/*N* = 4/1 CTR) showing the time course of the ISO effect on systolic Ca. **(C)** Averaged characteristics of cytoplasmic and nucleoplasmic CaTs. ^∗^*P* < 0.05, ^∗∗^*P* < 0.01, ^∗∗∗^*P* < 0.001, *n*/*N* = 30/7.

After basal recordings at 1.5 mM Ca, superfusion was switched to a recording buffer containing 10 nM ISO. Figure 2A shows original line-scan images and corresponding fluorescence traces of CaTs under control conditions (CTR, left panel) and 5 min after switching to ISO (right panel). Upon ISO stimulation, we observed a marked increase in both cytoplasmic and nucleoplasmic systolic fluorescence signals, whereas diastolic fluorescence was decreased. In addition, both compartments (Cyto and Nuc) showed a faster CaT decline.

We performed pilot experiments (*n*/*N* = 3/3), giving us a time course of the ISO effect on CaTs (Figure 2B) to determine an appropriate time point for further comparisons. These experiments showed that after 5 min exposure to ISO the effect on the systolic Ca level reached a maximum. This early time point seemed to be preferable for analysis as the occurrence of arrhythmic events could be observed less frequently compared to later time points. By contrast, in untreated control myocytes (CTR, *n*/*N* = 4/1), systolic Ca remained fairly constant over the 12 min observation period.

Comparison of average CaT characteristics (Figure 2C) indicates a substantial increase in systolic cytoplasmic Ca (Ca_cyto_) as well as in nucleoplasmic Ca (Ca_nuc_) in the presence of ISO. The systolic Ca_cyto_ was increased by 72% (from 5.31 ± 0.29 to 9.11 ± 0.50 F/F_rest_, *P* < 0.001; *n*/*N* = 30/7) while systolic Ca_nuc_ showed an increase by 58% (from 3.99 ± 0.20 to 6.29 ± 0.33 F/F_rest_, *P* < 0.001; *n*/*N* = 30/7). Moreover, we found diastolic Ca to be decreased in both cytoplasm and nucleoplasm (by 17% in cyto, from 1.48 ± 0.06 to 1.27 ± 0.06 F_0_/F_rest_, *P* < 0.01; *n*/*N* = 30/7 and by 13% in nuc, from 1.69 ± 0.09 to 1.50 ± 0.08 F_0_/F_rest_, *P* < 0.05; *n*/*N* = 30/7).

Also, kinetic parameters of CaTs were affected by ISO exposure. We found significantly shorter TTP in the nucleoplasm (TTP_nuc_, from 187 ± 11 to 137 ± 10 ms, *P* < 0.01; *n*/*N* = 30/7), whereas TTP_cyto_ showed an opposing trend (from 43 ± 4 to 50 ± 10 ms, *P* = 0.066; *n*/*N* = 30/7). Consistent for both compartments was an acceleration of CaT decay evident as a decrease of τ values by ∼40% (τ_cyto_ from 416 ± 29 to 249 ± 26 ms, *P* < 0.001, and τ_nuc_ from 773 ± 46 to 446 ± 45 ms, *P* < 0.001; *n*/*N* = 30/7).

The observed increase and the faster decay kinetics of cytoplasmic CaTs are presumably attributable to PKA- and CaMKII-mediated increases in trigger Ca and enhanced SERCA activity, respectively. The nuclear Ca, on the other hand, seemed to be affected in a very similar way. This might be explained by the cytoplasmic Ca increase in systole, the accelerated cytoplasmic CaT decline and increased SERCA activity in the NE.

### Modulation of Nuclear Ca Handling by SERCA Inhibition

The previous sets of experiments indicate, that the nucleoplasmic CaT follows changes of the cytoplasmic CaT over a wide range of Ca concentrations by yet not fully understood mechanisms. One factor may be passive diffusion of Ca through nuclear pores, whereas a direct or indirect involvement of Ca handling proteins must also be considered. We assumed the ISO-induced effects to be dependent, in part, on SERCA stimulation, as suggested by the profound acceleration of CaT decline. To further elucidate the role of SERCA for nuclear Ca regulation we used thapsigargin (TG), a specific and irreversible inhibitor of SERCA.

**FIGURE 3:**
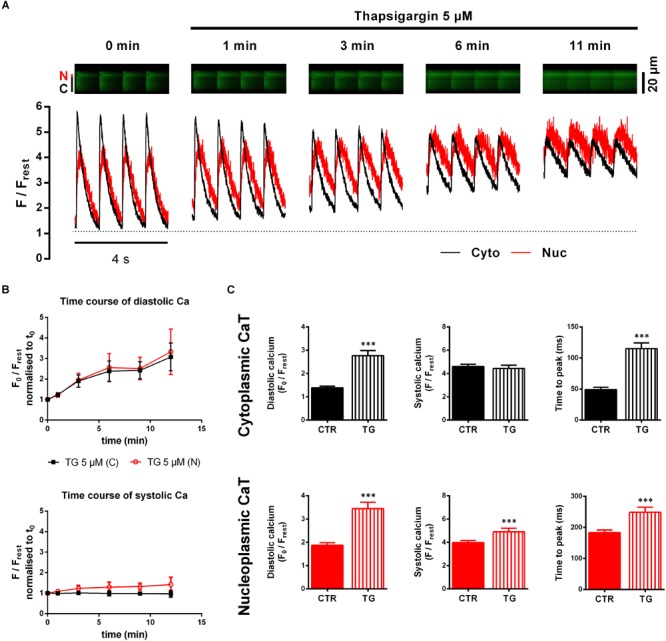
Cyto- and nucleoplasmic CaTs during SERCA inhibition by thapsigargin (TG). **(A)** Line-scan recordings of electrically stimulated cytoplasmic (C, Cyto, black) and nucleoplasmic (N, Nuc, red) CaTs at different time points after TG application. Fluorescence images (upper panel) and corresponding traces (lower panel) show a time-dependent increase in baseline fluorescence (=diastolic Ca) and slowing of decay kinetics. **(B)** Pilot experiments to study the time course of the TG effect (*n*/*N* = 5/1). Mean values of diastolic Ca (upper panel) normalized to *t*_0_ show a constant increase over time, whereas systolic Ca (lower panel) only increases in the nucleus. **(C)** Comparison of average CaT characteristics for the cyto- and nucleoplasm in the absence and presence of TG. ^∗∗∗^*P* < 0.001 vs CTR, *n*/*N* = 39/13.

Ventricular myocytes were superfused with recording buffer containing TG (5 μM). Figure 3A illustrates original fluorescence images and corresponding traces obtained before and during TG application at different time points. As exposure time progressed a continuous increase in diastolic fluorescence in both cytoplasm and nucleoplasm was observed, whereas at the same time systolic Ca only rose in the nucleus. In our hands, cytoplasmic CaT amplitudes were diminished as well, while, at the same time, systolic Ca_cyto_ levels were not significantly altered. Moreover, decay kinetics were markedly slowed down and changed from exponential to a rather linear decay (at 11 min). Under these conditions, with SERCA activity blocked, sarcolemmal Na/Ca exchange (NCX) will become the most important mechanism for Ca extrusion from the cell (Bers, 2002). Since NCX-mediated Ca extrusion is much slower than SERCA-mediated Ca decline [i.e., rate constants for NCX-mediated Ca extrusion are about 10 times lower than for SERCA-mediated Ca decline (Mederle et al., 2016)], decay kinetics appear linear in the first seconds. An initially recorded small set of cells (*n*/*N* = 5/1) served to study the time course (Figure 3B) of the TG effect in order to determine a suited time point for further analyses. The recordings were limited to a time (between 12 and 18 min) where effects of TG reached a maximum resulting in very high diastolic Ca concentrations and lack of CaTs, leaving the cells unable to contract, consistent with previous findings (Kirby et al., 1992; Wrzosek et al., 1992). Considering increasing cytoplasmic diastolic Ca fluorescence as a surrogate parameter for the strength of the TG effect, we opted for a time point of half maximal effect, which we found within the range of 2–7 min after TG application. Average time for analysis of the TG effect of all cells studied amounted to 3.5 ± 0.2 min (*n*/*N* = 39/13).

Figure 3C summarizes the results with TG. A doubling of diastolic Ca_cyto_ (from 1.38 ± 0.07 to 2.77 ± 0.22 F_0_/F_rest_, *P* < 0.001; *n*/*N* = 39/13) was found. Similarly, the nucleus showed significantly higher diastolic Ca levels after TG treatment (from 1.87 ± 0.11 to 3.45 ± 0.27 F_0_/F_rest_, *P* < 0.001; *n*/*N* = 39/13). TTP was slowed down in both compartments (TTP_cyto_ from 49 ± 4 to 115 ± 9 ms, *P* < 0.001, and TTP_nuc_ from 183 ± 9 to 249 ± 16 ms, *P* < 0.001; *n*/*N* = 39/13). In contrast, we observed for systolic Ca no changes in the cytoplasm, but a significant increase in the nucleoplasm. CaT decay was also impaired by TG. τ of decay, however, could not be analyzed because in the presence of TG decay was linear. In summary, TG elicited a large increase in diastolic Ca levels and impairment of CaT decay in both compartments.

### Relationship Between Cyto- and Nucleoplasmic Ca in Diastole

In order to analyze the relationship between cyto- and nucleoplasmic Ca, we plotted nucleoplasmic Ca as a function of cytoplasmic Ca (nuc-to-cyto plot) for both diastolic and systolic Ca.

**FIGURE 4:**
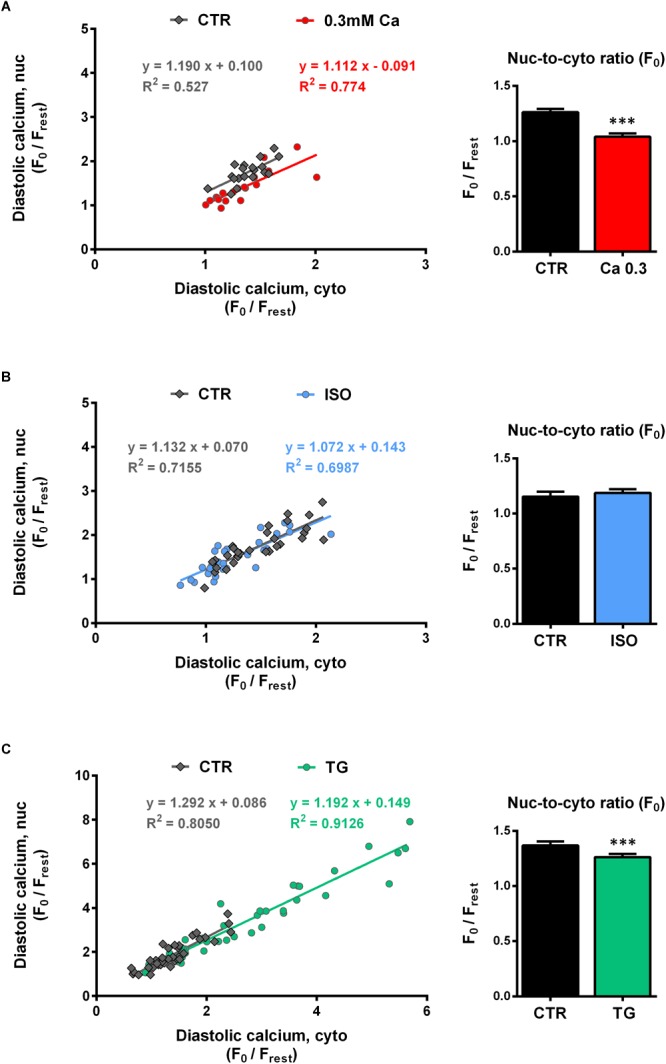
Diastolic nucleoplasmic vs cytoplasmic Ca. [**(A–C)**, left panels] Nuclear diastolic Ca as a function of cytoplasmic diastolic Ca. Correlation analysis revealed strong positive correlation for all groups with correlation coefficients *r* of 0.672 (CTR, 0.3 Ca), 0.853 (0.3 Ca, red), 0.867 (CTR, ISO), 0.824 (ISO, blue), 0.889 (CTR, TG) and 0.956 (TG, green); all *P* < 0.01. Individual values from the various groups (red = Ca 0.3, blue = ISO, green = TG, black = CTR) were fitted by a linear regression line (as indicated). [**(A–C)**, right panels] Average values of nuc-to-cyto ratios (N/C) of the diastolic Ca. Significantly decreased diastolic N/Cs were observed at 0.3 mM Ca (red) and after TG application (green); stimulation with ISO (blue) did not produce any significant changes. ^∗∗∗^*P* < 0.001 vs CTR, Ca 0.3: *n*/*N* = 19/7, ISO: *n*/*N* = 30/7, TG: *n*/*N* = 39/13.

Figure 4A–C (left panels) show the nuc-to-cyto plots for the diastolic Ca constructed from the experiments described above. There was a strong positive correlation between the diastolic Ca in the cytosol and in the nucleus for all groups investigated. The data points under CTR conditions (gray) as well as under experimental conditions (red = Ca 0.3, blue = ISO, green = TG) follow a linear relationship in all three experimental series with similar slopes between ∼1.1 and 1.3. At 0.3 mM Ca (Figure 4A, left), a downward shift in *y*-values was found, which corresponds to the selectively decreased nucleoplasmic diastolic Ca at 0.3 mM Ca (Figure 1C). The comparison of the slopes yielded no significant difference (Ca 0.3: 1.112 vs CTR: 1.190, *P* = 0.825). During ISO stimulation the mean diastolic Ca values were also decreased. The distribution of the diastolic Ca values, however, was not altered (Figure 4B, left) and the slopes were not significantly different (ISO: 1.072 vs CTR: 1.132, *P* = 0.753). During TG treatment high diastolic Ca values were added to the plot (Figure 4C, left) resulting in a distribution over a wide range of diastolic Ca values and a linear regression line with a similar slope as under CTR (TG: 1.192 vs CTR: 1.292, *P* = 0.523). Taken together, the nuc-to-cyto plots indicate that the diastolic nucleoplasmic Ca follows the diastolic cytoplasmic Ca in a linear fashion in all three experimental series.

The nuc-to-cyto ratio (N/C) represents the ratio of nucleoplasmic to cytoplasmic Ca and has been calculated as F_nuc_/F_cyto_ for systolic and diastolic Ca, respectively. Figure 4A–C (right panel) display the nuc-to-cyto ratio for the diastolic Ca. The diastolic Ca is typically found to be higher in the nucleus than in the cytoplasm which leads to a N/C > 1 (within all CTR experiments we found ratios between ∼1.1 and 1.4). At 0.3 mM Ca (Figure 4A, right), the diastolic N/C showed a significant decrease by 18% (from 1.26 ± 0.03 to 1.04 ± 0.03 F_0,nuc_/F_0,cyto_, *P* < 0.001; *n*/*N* = 19/7). Similarly, the diastolic N/C after TG exposure was decreased by 8% (from 1.37 ± 0.04 to 1.26 ± 0.03 F_0,nuc_/F_0,cyto_, *P* < 0.001; *n*/*N* = 39/13). Finally, no significant change in the diastolic N/C (from 1.15 ± 0.05 to 1.19 ± 0.03 F_0,nuc_/F_0,cyto_, *P* = 0.474; *n*/*N* = 30/7) was observed upon stimulation with ISO (Figure 4B, right).

### Relationship Between Cyto- and Nucleoplasmic Ca in Systole

**FIGURE 5:**
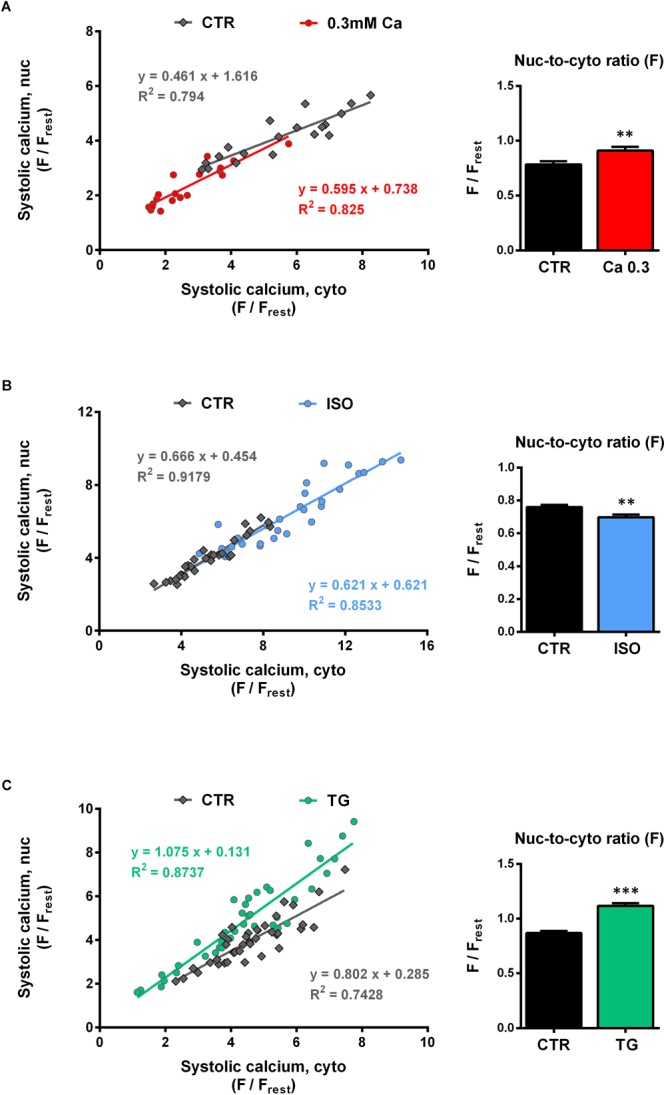
Systolic nucleoplasmic vs cytoplasmic Ca. [**(A–C)**, left panels] Nuclear systolic Ca as a function of cytoplasmic systolic Ca. Correlation analysis revealed strong positive correlation for all groups with correlation coefficients *r* of 0.867 (CTR, 0.3 Ca), 0.874 (0.3 Ca, red), 0.956 (CTR, ISO), 0.931 (ISO, blue), 0.830 (CTR, TG) and 0.913 (TG, green); all *P* < 0.001. Individual values from the various groups (red = Ca 0.3, blue = ISO, green = TG, black = CTR) were fitted by a linear regression line (as indicated). [**(A–C)**, right panel] Average values of nuc-to-cyto ratios (N/C) of the systolic Ca. Significant increases in systolic N/Cs were recorded at 0.3 mM Ca (red) and after TG application (green), whereas exposure to ISO (blue) reduced the N/C significantly. ^∗∗^*P* < 0.01, ^∗∗∗^*P* < 0.001 vs CTR, Ca 0.3: *n*/*N* = 19/7, ISO: *n*/*N* = 30/7, TG: *n*/*N* = 39/13.

Figure 5A–C (left panel) depict the nuc-to-cyto plots for the systolic Ca. Similar to the findings for the diastolic Ca, there was a strong positive correlation between the systolic Ca in the cytosol and in the nucleus for all groups investigated. The data points of CTR (gray) as well as of the three experimental interventions (red = Ca 0.3, blue = ISO, green = TG) were well described by linear functions. At 0.3 mM Ca (Figure 5A, left), systolic Ca values were shifted to the lower left of the graph, i.e., to lower cyto- and nucleoplasmic values. The slope at 0.3 mM Ca did not significantly differ from CTR (Ca 0.3: 0.595 vs CTR: 0.461, *P* = 0.148). By the use of ISO (Figure 5B, left) the range of values could be extended to very high Ca concentrations, where still a linear relationship between cyto- and nucleoplasmic Ca with a slope comparable to CTR (ISO: 0.621 vs CTR: 0.666, *P* = 0.558) could be observed.

For the TG data (Figure 5C, left), there was also a linear relationship between cyto- and nucleoplasmic Ca. In this case, the slope was significantly steeper compared to CTR (TG: 1.075 vs CTR: 0.802, *P* < 0.05), indicating a shift toward higher nucleoplasmic Ca levels at a given systolic cytoplasmic Ca concentration. Taken together, in line with the findings for the diastolic Ca, the systolic nuc-to-cyto plots suggest, that the systolic nucleoplasmic Ca follows the systolic cytoplasmic Ca in a linear fashion over a wide range of Ca concentrations.

The systolic nuc-to-cyto ratio serves as an indicator for the fraction of the cytoplasmic Ca that is transmitted to the nucleus during each contraction. In our experiments, we found the systolic N/C to vary between 0.70 and 0.85 under control conditions. At 0.3 mM Ca, the systolic N/C was significantly increased (Figure 5A, right) by 17% (from 0.78 ± 0.03 to 0.91 ± 0.04 F_nuc_/F_cyto_, *P* < 0.01; *n*/*N* = 19/7) and during TG exposure (Figure 5C, right) by 29% (from 0.87 ± 0.02 to 1.12 ± 0.03 F_nuc_/F_cyto_, *P* < 0.001; *n*/*N* = 39/13). ISO stimulation (Figure 5B, right), however, resulted in a decrease of the systolic N/C by 9% (from 0.76 ± 0.01 to 0.70 ± 0.02 F_nuc_/F_cyto_, *P* < 0.01; *n*/*N* = 30/7). In summary, the results imply that at 0.3 mM Ca and during TG treatment the propagation of systolic Ca from the cytoplasm into the nucleus is facilitated, whereas it is impaired in the presence of ISO.

### SERCA Function Affects Nuc-to-Cyto Ratios

The opposing effects on N/C of the three experimental interventions may be explained by a single mechanism, that is either inhibited in one case (at 0.3 mM Ca and during TG exposure) or stimulated in the other case (with ISO). We hypothesized, that SERCA activity may be this mechanism. This is supported by the fact, that TG as specific irreversible inhibitor of SERCA showed the most prominent effect on N/C.

The SERCA pump is responsible for the removal of Ca during diastole and contributes a fraction of ∼92% to the CaT decay in rat cardiac myocytes (Bers, 2002). In our experiments, the CaT decay is characterized by the τ of decay, which can be used as an indicator for SERCA activity. It has been shown previously that cytosolic CaT decay is slowed down during SERCA inhibition by TG (Kockskämper et al., 2001). This was also evident in our experiments (Figure 3A).

**FIGURE 6:**
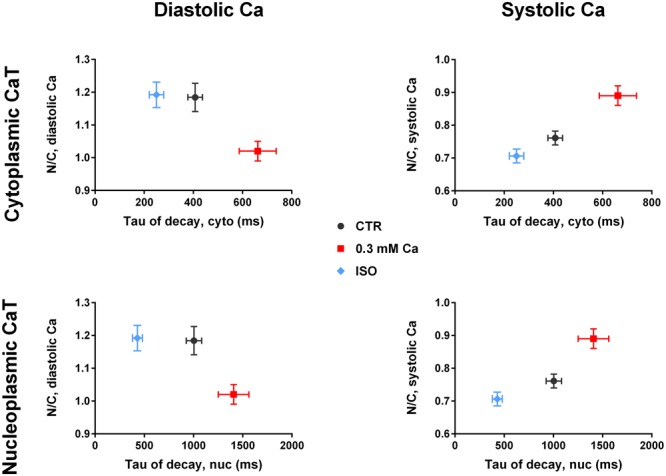
Nuc-to-cyto ratios vs tau of decay. (Left panel) Diastolic N/C as a function of τ of decay of the cytoplasmic CaT (top) and of the nucleoplasmic CaT (bottom). Diastolic N/C decreases with increasing τ. (Right panel) Systolic N/C as a function of τ of decay of the cytoplasmic CaT (top) and of the nucleoplasmic CaT (bottom). Systolic N/C increases with increasing τ. Ca 0.3: *n*/*N* = 19/7, ISO: *n*/*N* = 30/7, CTR: *n*/*N* = 26/10.

To further test our hypothesis we plotted N/C as a function of τ (Figure 6). For the diastolic Ca in cytoplasm (Figure 6, upper left panel) a higher value of τ at 0.3 mM Ca (red) was correlated with a decrease in N/C, whereas a lower value of τ under ISO stimulation (blue) did not alter the N/C. The same relation between N/C and τ was found in the nucleus (Figure 6, lower left panel). The plot for the systolic Ca (Figure 6, upper right panel) showed increased τ values at 0.3 mM Ca corresponding to a higher N/C. ISO decreased τ values corresponding to a lower N/C. Again, the same relation between N/C and τ was found in the nucleus (Figure 6, lower right panel).

For systolic Ca, the plot of N/C vs τ in Figure 6 (right panel) demonstrates an almost linear relationship in both cytoplasm and nucleus. This supports the hypothesis of SERCA activity directly affecting the N/C with higher activity (lower τ) reducing the N/C.

### Characterisation of Intracellular Ca Store Load and Ca Release

**FIGURE 7:**
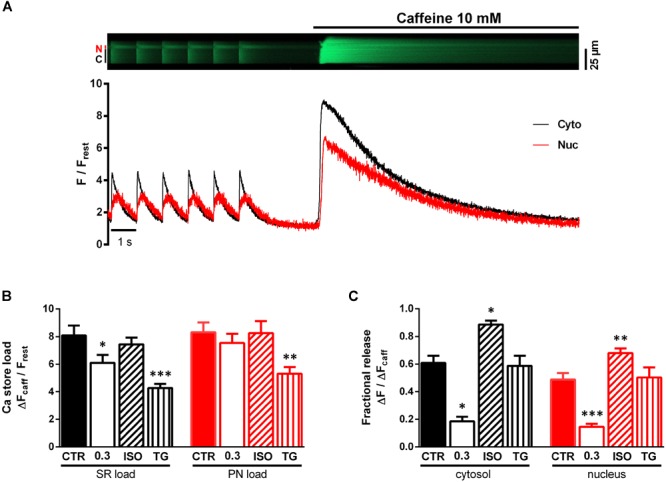
Characterisation of SR and PN store load and fractional release (FR). **(A)** Representative original line-scan recording and corresponding normalized fluorescence trace, showing electrically evoked cytoplasmic (C, Cyto, black) and nucleoplasmic (N, Nuc, red) CaTs followed by a stop of stimulation and rapid caffeine (10 mM) application producing a caffeine-induced CaT. **(B)** Bar graph of average Ca store loads, demonstrating significantly decreased SR loads (black) at 0.3 mM Ca and after treatment with TG. Additionally, TG induced a reduction of PN load (red). **(C)** Bar graph of average FR values. FR was decreased at 0.3 mM Ca and increased under ISO stimulation. TG did not alter FR. ^∗^*P* < 0.05, ^∗∗^*P* < 0.01, ^∗∗∗^*P* < 0.001 vs CTR, Ca 0.3: *n*/*N* = 9/5, ISO: *n*/*N* = 13/4, TG: *n*/*N* = 8/2, CTR: *n*/*N* = 9/6.

Since the function of SERCA is the active Ca transport into the SR and perinuclear (PN) stores, we considered changes in Ca store load and/or fractional Ca release from these stores to be involved in the observed effects on the nucleoplasmic CaT. To elucidate the role of the NE as Ca storage and release compartment on the nucleoplasmic CaT we used rapid caffeine application to deplete the SR and PN stores. Figure 7A illustrates an original line-scan image and corresponding normalized fluorescence traces from a caffeine application under control conditions. The myocyte was first paced at 1 Hz under control conditions (1.5 mM Ca) to evoke cytoplasmic (C, Cyto, black) and nucleoplasmic (N, Nuc, red) CaTs. The stimulation was then stopped, followed by a fast application of caffeine (10 mM), triggering a rapid depletion of Ca stores and, thus, a caffeine-induced CaT (CaT_caff_) in both cytoplasm and nucleus. The cytoplasmic CaT_caff_ represents the release of the SR Ca content, whereas the nucleoplasmic CaT_caff_ corresponds to the PN Ca content. The latter can be inferred from the fact that ventricular myocyte nuclei express RyRs and that caffeine is able to release Ca from the NE in isolated myocytes as well as in isolated nuclei (Zima et al., 2007; Ljubojevic et al., 2014; Plačkić et al., 2016).

In order to characterize the Ca load of the SR and PN we determined the amplitudes of the CaT_caff_ normalized to resting fluorescence (ΔF_caff_/F_rest_). Figure 7B shows a comparison of the SR Ca load (black) and PN Ca load (red) under the various experimental conditions. At 0.3 mM Ca SR Ca load decreased by 25% (from CTR of 8.08 ± 0.72 to 6.10 ± 0.57 ΔF_caff_/F_rest_ at Ca 0.3, *P* < 0.05; CTR: *n*/*N* = 9/6, Ca 0.3: *n*/*N* = 9/5) compared to CTR, but there was no change in PN Ca load. ISO stimulation left the SR Ca load unaltered, which is consistent with previous findings (Karpinski and Györke, 2001), and also did not affect PN Ca load. Upon TG application, there was a decrease in SR Ca load by 30% (to 4.27 ± 0.29 ΔF_caff_/F_rest_, *P* < 0.001; *n*/*N* = 8/2) as well as in PN Ca load by 29% (from CTR of 7.53 ± 0.67 to 5.31 ± 0.48 ΔF_caff_/F_rest_ under TG, *P* < 0.01; CTR: *n*/*N* = 9/6, TG: *n*/*N* = 8/2). Taken together these results show, that at 0.3 mM Ca and after TG exposure SR Ca load decreased significantly, presumably due to reduction of SERCA activity, resulting in a net loss of Ca from the SR. PN Ca load was only decreased by TG. Moreover, ISO exposure did not lead to any changes in both SR and PN Ca load.

The fractional release (FR) constitutes the fraction of Ca released from the Ca store content during a single contraction. It can be calculated as the ratio of the CaT amplitude and the amplitude of the CaT_caff_ (ΔF/ΔF_caff_). Under control conditions (CTR), FR amounted to ∼0.6 in the cytoplasm and ∼0.5 in the nucleus (Figure 7C), similar to values reported previously (Kockskämper et al., 2008a; Plačkić et al., 2016).

There was a large decrease of FR at 0.3 mM Ca by 69% in the cytoplasm (from CTR of 0.61 ± 0.05 to 0.19 ± 0.03 ΔF/ΔF_caff_ at Ca 0.3, *P* < 0.05; CTR: *n*/*N* = 9/6, Ca 0.3: *n*/*N* = 9/5) and also by 69% in the nucleoplasm (from CTR of 0.49 ± 0.05 to 0.15 ± 0.02 ΔF/ΔF_caff_ at Ca 0.3, *P* < 0.001; CTR: *n*/*N* = 9/6, Ca 0.3: *n*/*N* = 9/5). ISO exposure, on the other hand, greatly increased FR by 51% in the cytoplasm (to 0.89 ± 0.03 ΔF/ΔF_caff_, *P* < 0.05; *n*/*N* = 13/4) and by 39% in the nucleoplasm (to 0.68 ± 0.03 ΔF/ΔF_caff_, *P* < 0.01; *n*/*N* = 13/4). Interestingly, TG did not alter FR at all (FR_cyto_ from CTR of 0.61 ± 0.05 to 0.58 ± 0.07 ΔF/ΔF_caff_ under TG, *P* = ns and FR_nuc_ from CTR of 0.49 ± 0.05 to 0.50 ± 0.29 ΔF/ΔF_caff_ under TG, *P* = ns; CTR: *n*/*N* = 9/6, TG: *n*/*N* = 8/2).

### The Role of the PN Ca Stores

**FIGURE 8:**
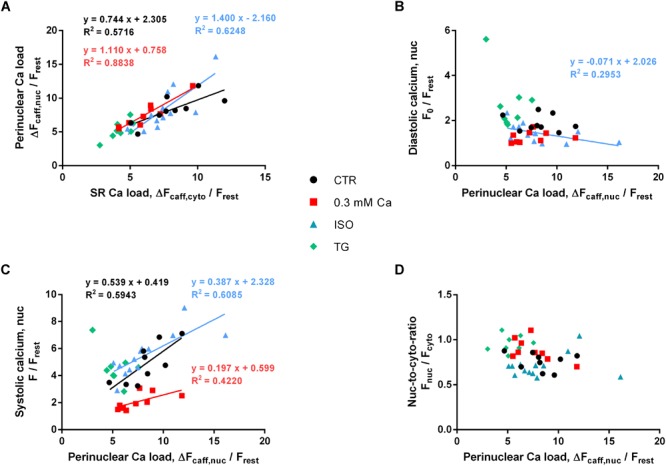
Role of PN Ca load. **(A)** PN Ca load as a function of SR Ca load. Correlation analysis revealed positive correlation for CTR, 0.3 Ca and ISO groups with correlation coefficients *r* of 0.850 (CTR, black), 0.900 (0.3 Ca, red) and 0.841 (ISO, blue); all *P* < 0.001. For TG, correlation was not significant (*P* = 0.069). Individual values from the three groups with positive correlation (black = CTR, red = Ca 0.3, blue = ISO) were fitted by a linear regression line (as indicated). **(B)** Diastolic Ca as a function of PN Ca load. Diastolic Ca did not correlate with PN Ca load (except for ISO; *r* = –0.632, *P* < 0.05). **(C)** Systolic Ca as a function of PN Ca load. Strong positive correlation was found for CTR, 0.3 Ca and ISO groups with correlation coefficients *r* of 0.733 (CTR, black, *P* < 0.05), 0.767 (0.3 Ca, red, *P* < 0.05) and 0.857 (ISO, blue, *P* < 0.001). Individual values from the three groups with positive correlation (black = CTR, red = Ca 0.3, blue = ISO) were fitted by a linear regression line (as indicated). **(D)** Systolic nuc-to-cyto ratio as a function of PN Ca load. No correlation between PN Ca load and systolic nuc-to-cyto ratio was found (*P* = ns for all groups). Ca 0.3: *n*/*N* = 9/5, ISO: *n*/*N* = 13/4, TG: *n*/*N* = 8/2, CTR: *n*/*N* = 9/6.

Figure 8A depicts PN Ca load as a function of SR Ca load. Individual values of the four groups (Ca 0.3, ISO, TG, CTR) are shown. There was a strong positive correlation between SR and PN Ca load for all groups, except for the TG group (*P* = 0.069). The distribution of the values implied a linear relationship between PN and SR Ca load, and linear regression analysis revealed slopes between 0.74 and 1.40 (as indicated). This dependence of PN Ca load on SR Ca load suggests, that the SR and the PN Ca stores represent one compartment of high interconnectivity, as reported previously (Wu and Bers, 2006).

In order to analyze the role of PN Ca load for the electrically stimulated CaT, we plotted diastolic and systolic Ca as a function of PN Ca load, as depicted in Figure 8B,C, respectively. As shown in Figure 8B, diastolic Ca did not appear to depend on PN Ca load, and there was no correlation between the two parameters in the CTR, 0.3 Ca and TG groups. Only the ISO group showed a negative correlation between diastolic Ca and PN Ca load (*P* < 0.05). Linear regression revealed a shallow negative slope of -0.071.

By contrast, systolic Ca appeared to increase with increasing PN Ca load in most groups (Figure 8C). Strong positive correlation was found for the CTR (*P* < 0.05), the 0.3 Ca (*P* < 0.05) and the ISO group (*P* < 0.001). Only the TG group did not show any correlation between systolic Ca and PN Ca load (*P* = 0.54). Slopes in the CTR, 0.3 Ca and ISO groups amounted to 0.539, 0.197 and 0.387, respectively. Finally, we tested whether the systolic nuc-to-cyto ratio (N/C) may depend on PN Ca load. As shown in Figure 8D, however, there was no correlation between these two parameters, suggesting that PN Ca load *per se* does not affect systolic Ca propagation into the nucleus.

## Discussion

The physiological and pathological relevance of nuclear Ca signaling in cardiac myocytes and its regulation have long been elusive. With recent studies showing nuclear Ca to play a major role in transcriptional control and—if impaired—to induce cardiac remodeling and hypertrophy (Ljubojevic et al., 2014; Ljubojevic and Bers, 2015; Plačkić et al., 2016; Dewenter et al., 2017; Olivares-Florez et al., 2018), it is important to elucidate the cellular mechanisms of nuclear Ca handling. In this study, we identify the cytoplasmic CaT and—as a novel regulator—SERCA activity as the major determinants of the systolic CaT in the nucleus, whereas modulation of PN Ca stores is much less important.

### The Cytoplasmic CaT Determines the Systolic Ca Increase in the Nucleus

We observed a strict dependence of systolic Ca in the nucleus on systolic Ca in the cytoplasm under all experimental conditions tested. Previous studies have found a similar dependence of nuclear CaTs on cytoplasmic CaTs (e.g., Kockskämper et al., 2008a; Ljubojevic et al., 2011). Here, we confirm and extend these findings by demonstrating that the linear dependence of systolic Ca in the nucleus on systolic Ca in the cytoplasm persists at both very low and very high Ca concentrations (Figure 5A–C). Thus, at reduced extracellular Ca of 0.3 mM, the decrease of the systolic Ca in the cytoplasm by ∼49% led to a decrease of systolic Ca in the nucleus by ∼56% (Figure 1C). After ISO application systolic Ca increased in the cytoplasm by 72% and at the same time in the nucleus by 58% (Figure 2C). These experiments, therefore, indicate that changes of systolic Ca in the cytoplasm are directly mirrored by the nuclear systolic Ca, implying a passive nature of the systolic Ca increase in the nucleus mediated mainly by diffusion of cytoplasmic Ca through nuclear pores. This notion is supported by the fact, that NPCs are readily permeable to Ca ions and that the nuclear CaT lags behind the cytoplasmic CaT with a delay. In addition, diffusion of Ca ions within the nucleus is significantly slower than in the cytoplasm (Soeller et al., 2003), which may contribute to the slower kinetics of nuclear CaTs.

In conclusion, there is a strict linear dependence of the nuclear systolic CaT on the cytoplasmic systolic CaT—ranging from very small Ca concentrations at 0.3 mM Ca to very high Ca concentrations under ISO stimulation—indicating that the cytoplasmic systolic Ca is the major determinant of the nuclear systolic Ca in cardiac myocytes.

### SERCA Limits the Systolic Ca Increase in the Nucleus

Increases in SERCA activity (accelerated CaT decay induced by ISO) impaired the systolic Ca increase in the nucleus. By contrast, decreases in SERCA activity (prolonged CaT decay in the presence of reduced extracellular Ca or thapsigargin) facilitated the Ca increase in the nucleus. Impairment (and facilitation) of nuclear Ca increases were evident as reduced (and increased) N/Cs as well as shallower (and steeper) slopes of the nuc-to-cyto plots. The latter effect was particularly pronounced in the presence of TG, the blocker of SERCA. Moreover, in the presence of TG, there was a selective increase in systolic nucleoplasmic Ca at unchanged systolic cytoplasmic Ca (Figure 3), providing direct evidence for the contribution of SERCA in limiting the systolic Ca increase in the nucleus.

**FIGURE 9:**
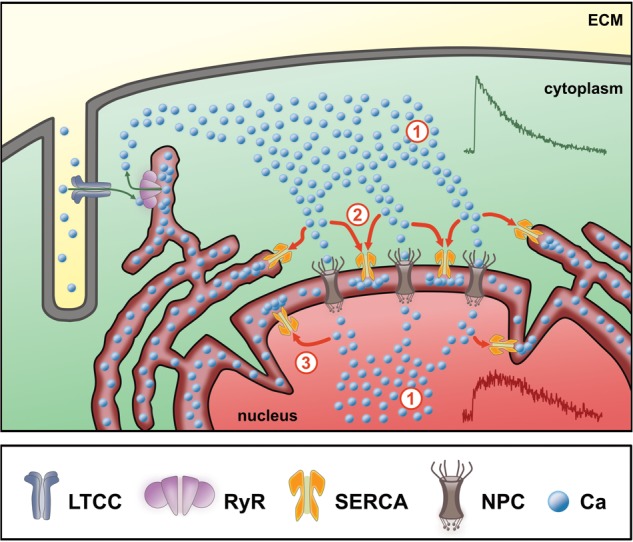
Scheme of nuclear Ca regulation in ventricular cardiomyocytes. During Ca-induced Ca release (green arrows) Ca from the extracellular matrix (ECM) enters the cytoplasm via L-type Ca channels (LTCC) into the junctional cleft between t-tubular membrane and sarcoplasmic reticulum (SR). Upon binding to ryanodine receptors (RyR) the SR releases stored Ca to cause the systolic Ca transient (CaT) in the cytoplasm (#1). The Ca gradient between the cytosol and the nucleus then drives the Ca ions through the nuclear pore complexes (NPC, #1). At this point, the magnitude of the cytoplasmic CaT (green CaT) represents the major determinant for the nucleoplasmic Ca increase (red). The Ca increase in the nucleus is further dependent on the activity of SERCA pumps (#2) located in the SR and nuclear envelope (perinuclear stores), which actively reduce the amount of free Ca reaching the nucleoplasm, hence limit the nucleoplasmic Ca increase. SERCA pumps located in the inner nuclear membrane (#3) may additionally contribute to modulate the systolic nuclear Ca increase.

Based on these results, we propose the following model for regulation of the systolic CaT in the nucleus of ventricular myocytes (Figure 9): the cytoplasmic systolic Ca increase is caused by CICR through RyRs located in the junctional SR. Cytoplasmic Ca ions then pass through NPCs in the NE and its invaginations—along their concentration gradient—to increase nuclear Ca (#1 in Figure 9). This leads to a delayed systolic Ca increase in the nucleus. SERCA pumps located in the NE and in the perinuclear SR can actively modify the local Ca concentration in the perinuclear area, thus affecting Ca propagation (#2 in Figure 9). SERCA may pump Ca back into the NE and SR, respectively, thus competing with NPCs and actively reducing the local Ca gradient between perinuclear cytoplasm and nucleoplasm. This process becomes more pronounced with higher SERCA activity (e.g., during stimulation of β-adrenergic receptors) resulting in reduced Ca propagation into the nucleus. On the other hand, when SERCA activity is reduced, Ca propagation into the nucleus is facilitated. Moreover, SERCA pumps located in the inner nuclear membrane may also contribute to modulate the nuclear systolic Ca increase directly from the nucleoplasm (#3 in Figure 9). The notion of SERCA pumps located in the NE controlling the nuclear CaT is backed up by ultrastructural data showing that SERCA and PLB are both found in the NE and its invaginations with SERCA being concentrated in the outer nuclear membrane (Ljubojevic et al., 2014; Wu et al., 2016). Moreover, the PLB-to-SERCA ratio is increased in the NE implying that PLB regulation of SERCA is particularly pronounced in the NE (Wu et al., 2016). This suggests that during β-adrenergic stimulation SERCA pumps located in the NE become stimulated to a larger extent than those located in the SR. In conclusion, SERCA can actively limit the nucleoplasmic Ca increase in systole and may thereby serve as a protective factor for maintaining nuclear Ca homeostasis by shielding the nucleus from excessive Ca increases during high sympathetic tone.

### No Major Role for PN Ca Stores

Our data suggest cytoplasmic Ca as major determinant of the nuclear systolic Ca. On the other hand, there is evidence that nuclear CaTs may be at least partly regulated independently from cytoplasmic CaTs (Wu et al., 2006; Kockskämper et al., 2008a,b; Ibarra et al., 2013). The nucleus harbors its own Ca stores, that can actively release Ca into the nucleoplasm via, e.g., IP_3_R Ca release channels located in the NE (Wu et al., 2006; Zima et al., 2007; Kockskämper et al., 2008a; Escobar et al., 2011; Ibarra et al., 2013; Chen et al., 2018). Moreover, sarcolemmal IGF-1 receptors in close proximity to the nucleus may exert local control on IP_3_-dependent nuclear Ca release independent of cytosolic Ca signaling (Ibarra et al., 2013). Finally, there is evidence for RyR-mediated Ca release from the NE in cardiac myocytes (Yang and Steele, 2005; Zima et al., 2007). Thus, it is feasible that the NE Ca store load and FR may modulate nuclear CaTs.

Plotting the PN Ca load as a function of the SR Ca load (Figure 8A) resulted in a linear relationship over a wide range of Ca loads. This suggests that NE Ca load follows SR Ca load (or *vice versa*) and indicates that these two intracellular Ca storage compartments are highly interconnected, as reported previously (Wu and Bers, 2006). During ISO stimulation SR Ca load was unchanged, as was PN Ca load. By contrast, TG reduced SR Ca load and, likewise, PN Ca load by a similar fraction. With both Ca store loads reduced to a similar proportion under these conditions it was surprising to find a systolic Ca increase exclusively in the nucleus (Figure 3C). This observation underpins the importance of SERCA activity for shielding the nucleus from excessive Ca increases during electrically stimulated CaTs. Interestingly, at 0.3 mM extracellular Ca SR Ca load was reduced, whereas PN Ca load remained unaltered (Figure 7B), although systolic Ca was decreased in both, cytoplasm and nucleus, to a similar degree. This suggests that, particularly at low fractional SR Ca release, the NE Ca store may lose less Ca and retain the stored Ca better than the SR.

There was a positive correlation between PN Ca load and systolic Ca in the nucleus under most experimental conditions (Figure 8C). This may indicate that PN Ca load is involved in regulation of systolic nuclear Ca. We suggest, however, that this is an indirect effect given that PN Ca load is dependent on SR Ca load and SR Ca load determines the amplitude of the cytosolic CaT, which, in turn, is the main determinant of the nuclear CaT. Several lines of evidence support the notion that PN Ca load is not a decisive factor for systolic nuclear Ca: (1) Reduction of extracellular Ca (to 0.3 mM Ca) produced a large decrease in systolic nuclear Ca, but left PN Ca load unchanged. (2) In contrast, ISO markedly increased systolic nuclear Ca, again without changing PN Ca load. Thus, systolic nuclear Ca could vary markedly at almost identical PN Ca loads (Figure 7B). (3) Moreover, decreasing the PN Ca store content with TG even increased systolic nuclear Ca. (4) Finally, the systolic nuc-to-cyto ratio was not dependent on PN Ca load (Figure 8D). Taken together, these findings suggest that alterations in PN Ca store load may play only a minor role in regulating the systolic nuclear CaT.

Fractional release from perinuclear Ca stores (FR_PN_) amounted to ∼50% under control conditions (Figure 7C), very similar to values reported previously for both atrial and ventricular myocytes (Kockskämper et al., 2008a; Plačkić et al., 2016). It was less than FR from SR Ca stores (FR_SR_) (Figure 7C), again consistent with previous observations (Kockskämper et al., 2008a; Plačkić et al., 2016). This was true for all experimental conditions tested (CTR, 0.3 Ca, ISO, TG). The fact that FR_PN_ was always less than FR_SR_ may be explained by the preferentially passive nature of the nuclear Ca increase in systole, i.e., Ca diffusion from cytoplasm through nuclear pores, with only a small contribution from active RyR-mediated Ca release from perinuclear/NE Ca stores (Yang and Steele, 2005; Zima et al., 2007). Moreover, perinuclear SERCA activity limits the perinuclear/nuclear Ca increase. Changes in FR_SR_ were consistently matched by similar changes in FR_PN_. This is true for reduced extracellular Ca concentration (0.3 mM Ca) and β-adrenergic stimulation (ISO). TG application, however, left FR from both Ca stores unaltered, but increased systolic Ca in the nucleus selectively. Thus, substantial differences in FR_PN_ were observed, ranging from <20% at 0.3 mM Ca to ∼65% in the presence of ISO (Figure 7C), while PN Ca load remained almost constant (Figure 7B). This clearly indicates, that PN Ca load *per se* does not regulate FR_PN_.

### Regulation of Diastolic Ca in the Nucleus

The diastolic Ca proved to be very susceptible to changes of SERCA function as the most important mechanism for CaT decline. This effect was particularly pronounced when SERCA was blocked by TG (Figure 3, 4). As for systolic Ca, there was also a linear relationship between diastolic Ca in the nucleus and in the cytoplasm (Figure 4, left panels) over a wide range of Ca concentrations with similar slopes of ∼1.1–1.3. It suggests that—as for systolic Ca—cytoplasmic Ca is a major determinant of nuclear Ca in diastole (or *vice versa*). In our experiments, the diastolic Ca was found to be higher in the nucleus than in the cytoplasm, which is in line with previous studies using calibrated Ca concentrations (Ljubojevic et al., 2011; Plačkić et al., 2016). Accordingly, the slopes of the nuc-to-cyto plots and the diastolic N/Cs exhibited values >1 (Figure 4). The higher diastolic Ca in the nucleus is caused mainly by the slower decay kinetics of the nuclear CaTs which prevent that a baseline Ca level as low as in the cytoplasm can be reached within the time period between two excitations (Ljubojevic et al., 2011).

The markedly slower CaT decay in the nucleus suggests, that the fast mechanisms for Ca decline in the cytoplasm, i.e., NCX and SERCA, are less available for nuclear Ca decline, even though the presence of these proteins in the NE has been shown. NCX was found to be located in the inner nuclear membrane and also in the region of the nuclear invaginations, from where it could pump Ca back into the NE lumen (Ledeen and Wu, 2007). SERCA was found in both the inner and outer nuclear membrane, although the major fraction of the protein was located in the outer nuclear membrane (Gerasimenko and Gerasimenko, 2004; Ljubojevic et al., 2014; Wu et al., 2016). During cytoplasmic CaT decline there is dynamic competition between NCX and SERCA. In rat ventricular myocytes, SERCA contributes a fraction of ∼92% to the cytoplasmic CaT decline and thereby exceeds the contribution of NCX (∼7%) by far (Bers, 2002). Assuming a similar contribution to nucleoplasmic CaT decay makes SERCA the most important re-uptake mechanism for nucleoplasmic Ca. With the major fraction of SERCA located in the outer nuclear membrane, it requires Ca to prior diffuse out of the nucleus through NPCs for a quantitatively relevant re-uptake into the perinuclear Ca stores. This may also account for the slower decay kinetics of the nucleoplasmic CaTs.

Diastolic nuclear Ca did not correlate with PN Ca load under most experimental conditions examined (Figure 8B). We observed a significant correlation of the two parameters only in the presence of ISO with a very shallow slope (Figure 8B). This suggests that the PN Ca load was no major factor for regulating diastolic nuclear Ca.

Taken together, these findings indicate that cytoplasmic diastolic Ca and SERCA function are the two most important factors regulating diastolic nuclear Ca. In addition, tonic Ca release (Ca leak) from perinuclear Ca stores might contribute to diastolic nuclear Ca regulation, as determined in a previous study (Ljubojevic et al., 2011).

### Limitations of the Study

We did not calibrate the Fluo-4 fluorescence values and, thus, we can not directly compare the Ca regulation in cytoplasm vs nucleus in terms of absolute Ca concentrations. It is known that the fluorescence properties of Fluo-4 differ between cytoplasm and nucleus with lower apparent K_d_ values for Ca binding in the nucleus (Ljubojevic et al., 2011; Plačkić et al., 2016). Hence, we can only report and quantify relative fluorescence changes in the two compartments, which have to be interpreted with some caution. For example, resting and diastolic Ca concentrations are higher in the nucleus than in the cytoplasm, implying that we underestimate nuclear Ca changes and the respective nuc-to-cyto ratios. Also, kinetics of CaT decline (but not time-to-peak) will change when considering the different Ca binding properties of Fluo-4 in cytoplasm vs nucleus. The differences between nuclear and cytoplasmic CaT decay (with the nucleus showing much slower decay), however, persist when using calibrated Ca concentrations (Ljubojevic et al., 2011). Moreover, we have tried to circumvent some of these limitations by conducting paired experiments (e.g., Figure 1–5). For example, when an intervention alters CaT decay or diastolic and systolic Ca, absolute changes after calibration would differ, but the direction of change will remain the same. Similarly, using absolute Ca concentrations for the nuc-to-cyto plots (Figure 4, 5) would change (i.e., increase) the steepness of the slopes, but not the finding that nuclear Ca depends on cytoplasmic Ca with higher nuclear values corresponding to higher cytoplasmic values. Thus, our major conclusions—dependence of nuclear Ca on cytoplasmic Ca and SERCA activity controlling the nuclear Ca increase in systole—will not be affected by using Fluo-4 fluorescence data rather than calibrated Ca concentrations.

## Conclusion

The present study shows that the systolic cytoplasmic Ca is the major determinant of the systolic nuclear Ca increase. In addition, it provides evidence that SERCA function directly modulates passive Ca propagation into the nucleus, thereby limiting the nuclear Ca increase in systole. Based on our results (Figure 9), we propose that: (1) the nucleoplasmic Ca increase in systole is caused to a large fraction and over a wide range of systolic Ca concentrations by passive diffusion of cytoplasmic Ca through nuclear pores; (2) propagation of Ca from the cytoplasm into the nucleus is controlled by SERCA, located on the NE and perinuclear SR, which limits the systolic Ca increase in the nucleus; (3) under physiological conditions PN Ca store load plays only a minor role in regulation of nuclear Ca.

At this time, we have only begun to understand the mechanisms regulating the complex nature of nuclear Ca signaling. Given the fact, that this versatile ion controls so many important cellular functions and, in addition, that it is a key player in a variety of pathological conditions will make nuclear Ca regulation an exciting topic for future research.

## Author Contributions

JK and TK conceived the research. TK performed the experiments and analyzed original data. TK and JK analyzed, discussed and interpreted the data. TK and JK drafted the manuscript. TK and JK approved the final version of the manuscript.

## Conflict of Interest Statement

The authors declare that the research was conducted in the absence of any commercial or financial relationships that could be construed as a potential conflict of interest.
